# A**poE Influences the Blood-Brain Barrier Through the NF-κB/MMP-9 Pathway After Traumatic Brain Injury**

**DOI:** 10.1038/s41598-017-06932-3

**Published:** 2017-07-27

**Authors:** Zhipeng Teng, Zongduo Guo, Jianjun Zhong, Chongjie Cheng, Zhijian Huang, Yue Wu, Shuang Tang, Chao Luo, Xing Peng, Haitao Wu, Xiaochuan Sun, Li Jiang

**Affiliations:** 1 Department of Neurosurgery, the First Affiliated Hospital of Chongqing Medical University, Chongqing, China; 2 Department of Neurosurgery, Chongqing Traditional Chinese Medicine Hospital, Chongqing, China; 3 Department of Neurosurgery, The Affiliated Hospital of Zunyi Medical College, Zunyi, Guizhou China; 4 Department of Neurosurgery, Suining Central Hospital, Suining, Sichuan China

## Abstract

Apolipoprotein E (ApoE), encoded by the ApoE gene (APOE), influences the outcomes of traumatic brain injury (TBI), but the mechanism remains unclear. The present study aimed to investigate the effects of different ApoEs on the outcome of TBI and to explore the possible mechanisms. Controlled cortical impact (CCI) was performed on APOEε3 (E3) and APOEε4 (E4) transgenic mice, APOE-KO (KO) mice, and wild type (WT) mice to construct an *in vivo* TBI model. Neurological deficits, blood brain barrier (BBB) permeability and brain edema were detected at days 1, 3, and 7 after TBI. The results revealed no significant differences among the four groups at day 1 or day 3 after injury, but more severe deficits were found in E4 and KO mice than in E3 and WT mice. Furthermore, a significant loss of tight junction proteins was observed in E4 and KO mice compared with E3 and WT mice at day 7. Additionally, more expression and activation of NF-κB and MMP-9 were found in E4 mice compared with E3 mice. Different ApoEs had distinct effects on neuro-function and BBB integrity after TBI. ApoE3, but not E4, might inhibit the NF-κB/MMP-9 pathway to alleviate BBB disruption and improve TBI outcomes.

## Introduction

Traumatic brain injury (TBI) is the leading cause of mortality and disability among young individuals, and the incidence of TBI is rising sharply around the world. Usually, TBI is divided into primary injury and secondary injury. Secondary injury after TBI includes a series of complex processes, such as an excessive release of excitotoxin, a disturbance of intracellular calcium levels, and an increased release of inflammatory mediators and oxidative factors. All of the above are associated with the disruption of the blood brain barrier (BBB) and subsequent brain edema, which has a crucial impact on disability and mortality after TBI^1^. Therefore, reducing brain edema by protecting BBB integrity urgently needs to be addressed in order to improve neuro-function after TBI.

Matrix metalloproteinase-9 (MMP-9) belongs to a family of zinc-binding proteolytic enzymes that remodel the extracellular matrix under normal conditions. In pathological conditions, MMP-9 plays an important role in the degradation of the major components of the basal lamina, particularly type IV collagen, laminin, and fibronectin^2, 3^. Furthermore, MMP-9 is involved in BBB disruption in ischemic injury and is associated with increased vasogenic edema and diffusion lesion volume^4^. In our previous study, the expression of MMP-9 was found to be greatly increased with laminin reduction and BBB rupture in hemorrhagic stroke, but laminin degradation and BBB rupture could be alleviated by inhibiting MMP-9^5–7^. In addition, MMP-9 appears to be down-regulated by nuclear factor-κB (NF-κB) suppression^8, 9^. Meanwhile, a recent study showed that MMP-9 might also influence this process after TBI by increasing capillary permeability and brain edema, both of which are considered typical symptoms of secondary injury after TBI^10^.

Apolipoprotein E (ApoE) is the most important apolipoprotein in the brain tissue. Three ApoE isoforms exist in humans, ApoE2, ApoE3 and ApoE4, which are encoded by three different alleles, APOE ε2, ε3, and ε4, respectively^11^. ApoE4, which is encoded by APOEε4, is considered a dysfunctional or harmful protein compared with ApoE2 and ApoE3. Moreover, ApoE4 is considered a negative factor that contributes to unfavorable outcomes in both Alzheimer’s disease (AD) and TBI^12, 13^. Our previous studies also showed that ApoE can influence the outcome of TBI, but the mechanism remains unknown^14–18^. Furthermore, the influence of ApoE on the BBB has been studied in some research, and our recent studies revealed that ApoE may protect BBB integrity after TBI^19–23^. ApoE was recently reported to play a protective role in autoimmune encephalomyelitis (EAE) by maintaining BBB integrity, which may occur through the inhibition of MMP-9 expression^24^. Robert *et al*.^13^ also found that the expression of ApoE4 and a lack of murine ApoE caused BBB breakdown by activating the NF-κB/MMP-9 pathway in pericytes.

However, the effects of different ApoEs on the BBB after TBI are not clear, and the relationship among ApoE, MMP-9 and BBB are poorly understood. In the present study, we investigated the effects of different ApoE isoforms on the BBB after TBI and the possible mechanism through which ApoE influences the BBB after TBI.

## Materials and Methods

All animal procedures were approved by the Experimental Ethics Committee at the First Affiliated Hospital of Chongqing Medical University in Chongqing, China. All experiments were performed in accordance with the experimental regulations of Chongqing Medical University.

### Animals

APOE transgenic mice, E3 and E4 mice, and APOE knock-out mice were obtained from the Medical Institute of Experimental Animals in Chinese Academy of Medical Sciences. Wild type mice (C57BL/6 J) were provided by the Experimental Animal Center of Chongqing Medical University. E3 and E4 mice transgenic mice respectively express human ApoE3 and ApoE4, which are detectable in the glial cells and neuropils of mice under the direction of the human glial fibrillary acidic protein (GFAP) promoter. None of these mice express mouse ApoE. The human ApoE3 and ApoE4 expression patterns follow the endogenous mouse ApoE and GFAP expression patterns in the brain.

All animals were 8–12 weeks old and weighed 18–26 g. They were divided into 4 groups: the E3 group, the E4 group, the KO group and the WT group.

### *In vivo* TBI model - Controlled Cortical Impact (CCI)

CCI was produced on the exposed cortex using a controlled impactor device, the TBI Model system (TBI 0310, Precision Systems and Instrumentation, USA), as previously described^23^. Briefly, the mice were deeply anesthetized, and then, a 5-mm right lateral craniotomy centered at 2.7 mm lateral from the midline and 3 mm anterior to the lambda was performed using a motorized drill. The skull was removed with intact dura mater. CCI was produced using a pneumatic cylinder with a 3-mm diameter flat-tip impounder at an impact velocity of 3 m/s, a dwell time of 100 ms, and a cortical contusion depth of 1.5 mm. A plastic skull cap was secured over the craniotomy after the injury, and the scalp was sutured. The rectal temperature was maintained at 37 ± 0.5 °C with a heating pad if required. The animals were observed for 30 min and returned to their cages with free access to food and water. A moderately severe contusion was produced in the right sensorimotor cortex with pronounced behavioral deficits but virtually no mortality.

### Rotarod Test

**Figure 1:**
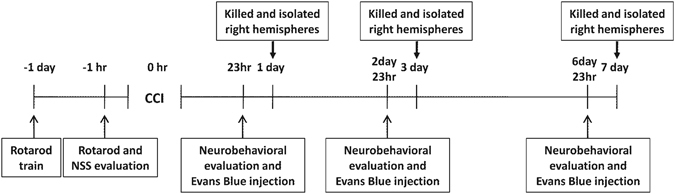
Schematic demonstrating the overall experimental process.

A rotarod instrument (ZB-200 Rota-Rod Treadmill, Taimeng Software Co. Ltd, Chengdu, China) was used to assess the motor function of mice by an investigator who was blind to the experimental groups. The animals underwent three trials at an interval of 15 min between the trials at a standard rotational speed of 16 rpm for 1 min on the day before CCI. Three trials with a rotational speed acceleration within 2 min were performed at 1 h before CCI. The average time to fall from the rotating cylinder in the three trials after CCI at days 1, 3, and 7 was recorded as the baseline latency (Fig. 1). The average latency of the time to fall from the rotarod device was recorded for each mouse.

### Neurological Severity Score (NSS)

Neurological deficits were evaluated at different time points by the NSS according to Table 1^25^ by an investigator blinded to the grouping. The NSS contained motor, sensory, reflex, and balance tests. Each animal was trained for the NSS items at 24 hours and 1 hour before CCI (Fig. 1), and the points for each task and the maximal score were obtained 1 hour before sacrifice. Neurological function was graded on a scale of 0 to 10 (0 = normal; 10 = maximal deficit) (Supplemental Table 1). The mice were allowed three attempts to fulfill the task.

### Blood-brain Barrier (BBB) Permeability

BBB permeability was detected by testing Evans Blue (EB) extravasation at day 1, day 3, and day 7 after TBI. First, the animals were anesthetized 1 hour before being killed and were injected intravenously with 2% EB in phosphate buffered saline (PBS) at a dose of 2 ml/kg. One hour later, they were re-anesthetized and perfused through the left cardiac ventricle with saline to clear intravascular EB dye. Then, the mice were decapitated, and the right hemispheres were removed and homogenized in 2 ml of PBS. One milliliter of trichloroacetic acid was added to precipitate protein, and the samples were centrifuged at 12,000 rpm for 15 min. The resulting supernatant was measured for the absorbance of EB at 630 nm using a spectrophotometer. The results were expressed as μg of EB/mg tissue.

### Brain Water Content

Brain edema was detected using the wet/dry method as previously described^6^. Briefly, the right hemispheres were rapidly removed from the skull, separately placed into pre-weighed and labeled glass vials, and weighed to obtain the wet weight. The vials were subsequently placed in an oven at 105 °C for 24 hours, followed by re-weighing to obtain the dry weight. The brain water content was calculated as [(wet weight − dry weight)/wet weight] × 100%.

### Matrix Metalloproteinase Zymography

The activity of MMP-9 was assessed at day 7 after TBI using the gelatin zymography method according to the instruction for the MMP gelatin-zymography electrophoretic analysis reagent kit (GENMED, China). The right hemisphere samples were homogenized in lysis buffer including protease inhibitors at 50 mg/ml and then centrifuged at 12,000 rpm for 15 min at 4 °C. The total protein concentration was determined by the BCA assay (Beyotime, China). The samples (40 μg per lane) were loaded and separated by 8% Tris-tricine gel with 0.1% gelatin as a substrate. After separation by electrophoresis, the gel was renatured for 1 h and then incubated with digestive buffer at 37 °C for 36 h. The gel was stained with 0.5% Coomassie Blue R-250 for 60 min and destained for 6–24 h until a clear white band appeared on a blue background. MMP-9 activity reflected by the white band was quantified by ImageJ.

### Western Blot

The injured cortex of the right hemisphere samples were mechanically homogenized in radioimmunoprecipitation assay (RIPA) lysis buffer (Beyotime). The lysates were centrifuged at 12,000 rpm for 15 min at 4 °C. The protein concentration was estimated using the BCA Protein Assay Kit (Beyotime). The samples (40 μg per lane) were separated by 10% SDS-PAGE and electro-transferred onto a polyvinylidene-difluoride membrane (Millipore, USA). The membrane was blocked with 5% bovine serum albumin (BSA) for 1 hour at room temperature and incubated overnight at 4 °C with primary antibodies directed against Occludin (Santa Cruz, USA), Zonula occluden-1 (ZO-1) (Santa Cruz), NF-κB (Cell Signaling, USA), I-κB (Proteintech, China), phosphor-I-κB (Cell Signaling) and MMP-9 (Abcam, USA) diluted in Primary Antibody Dilution Buffer (Beytime) at 1:500 (Occludin, ZO-1, I-κB) or 1:1000 (NF-κB, MMP-9). β-actin (diluted in 1:1000; Abcam) was used as a loading control. After the membrane was washed for 10 min three times in PBS + Tween 20 (PBST), it was incubated with the appropriate horseradish peroxidase (HRP)-conjugated secondary antibody (diluted 1:3000 in PBST; Beyotime) for 1 h. The densitometry analysis was performed with the Chemi-Doc detection system and Quantity One software (Bio-Rad, USA).

### Statistical Analysis

All data were presented as the means ± standard deviation. SPSS 17.0 was used to analyze the data between the groups with a one-way analysis of variance (ANOVA) test followed by the Least Significant Difference (LSD) test. Statistical significance was inferred at *P* < 0.05.

## Results

### Neurological deficits

**Figure 2:**
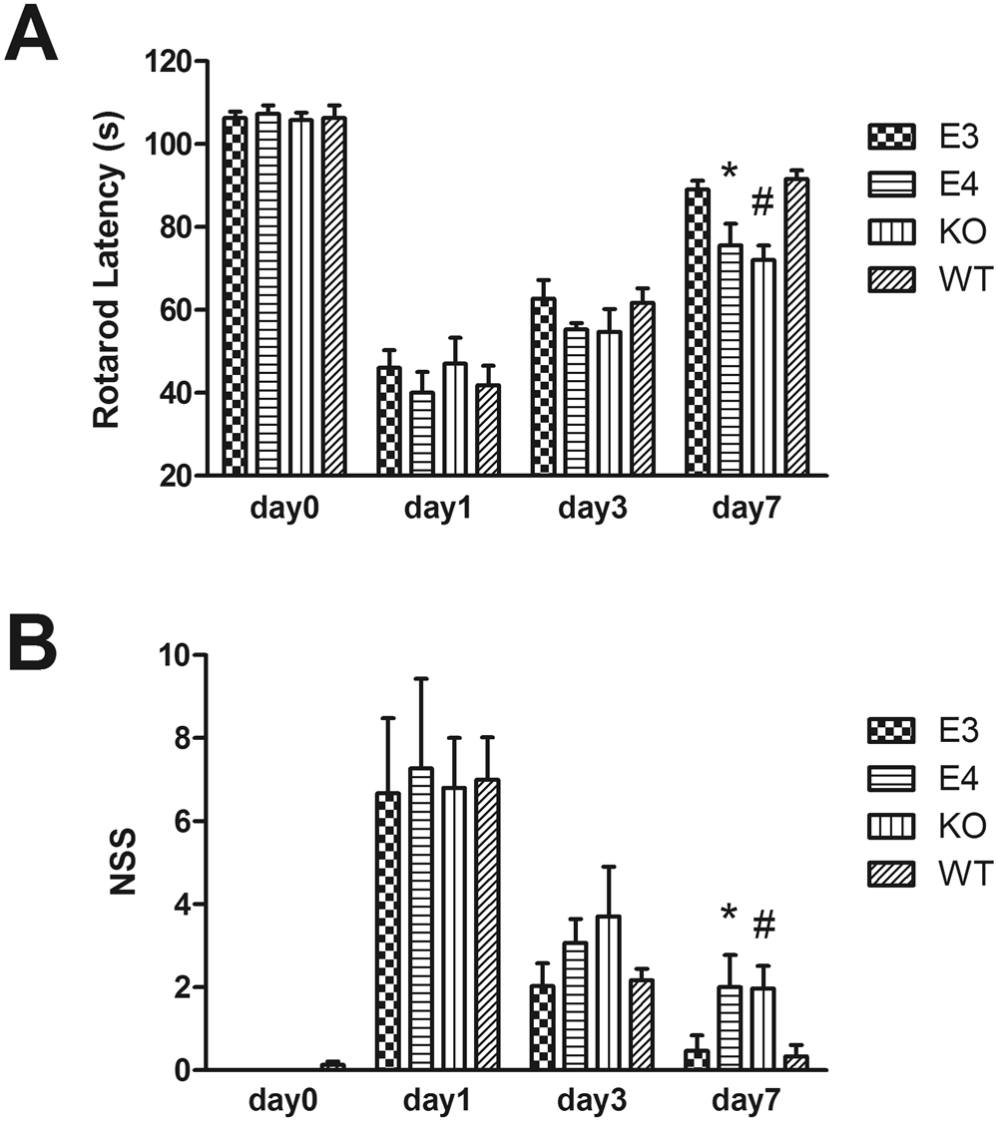
Neurobehavioral status after CCI was evaluated by the Rotarod and NSS methods. Obvious neurological deficits were observed at day 1 and day 3 after TBI. However, at day 7, the WT mice recovered significantly more than the E4 and KO mice, and the E3 mice recovered faster than the E4 mice. (*P < 0.05 vs E3 mice and WT mice; ^#^ *P* < 0.01 vs WT mice. N = 8). The data are presented as the mean ± SD.

The Rotarod test and NSS results showed that obvious neurological deficits appeared following injury (Fig. 2A and B), with the maximum injury appearing at day 1. At day 1 and day 3 after injury, no significant differences were found among E3, E4, KO and WT groups, but the statuses of individual mice differed at day 7. The Rotarod test revealed more impaired motor function in the KO and E4 groups compared to the WT group at day 7 after injury (*P* < 0.01 vs KO, and *P* < 0.05 vs E4), and E3 mice showed better motor function than E4 mice (*P* < 0.05). The NSS evaluation also revealed more impaired neural function in the KO group and the E4 group compared to the WT group at day 7 after injury (*P* < 0.01 vs KO, and *P* < 0.05 vs E4), and the E3 mice showed better neural function compared with the E4 mice (*P* < 0.05). Both the Rotarod test and the NSS results showed that the mice in the E4 and KO groups suffered more serious neurological deficits compared with the mice in the E3 and WT groups, indicating that the neuro-function of E4 and KO mice recovered more slowly. However, no significant difference was found between the E3 group and the WT group (*P* > 0.05).

### BBB Permeability

**Figure 3:**
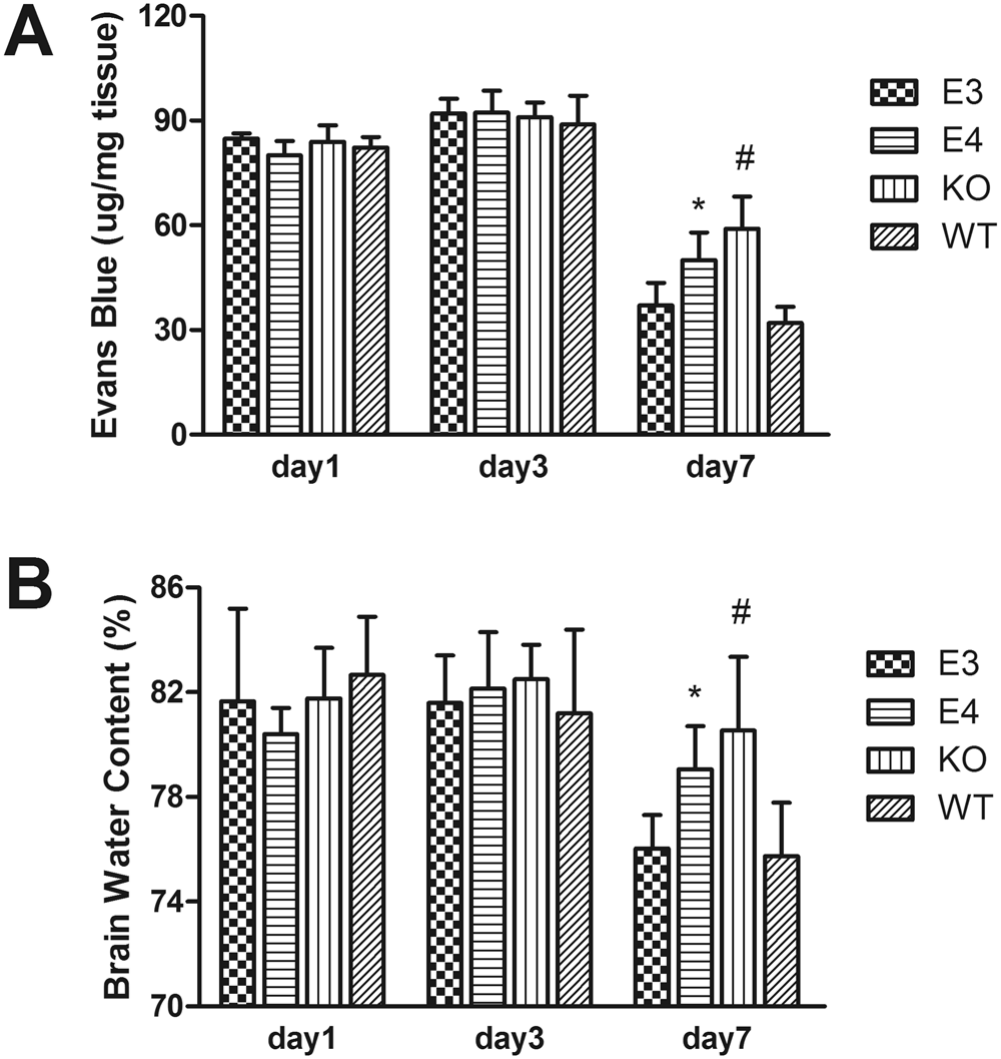
(**A**) BBB permeability was detected by EB extravasation after TBI, and no significant differences were observed in each group at day 1 or day 3. However, EB dye exudation significantly decreased in each group at day 7 and decreased more sharply in E3 and WT mice. (*P < 0.05 vs E3 mice and *P* < 0.01 vs WT mice; ^#^ *P* < 0.01 vs WT mice. N = 8) (**B**) The brain water content significantly increased after injury. Consistent with the BBB permeability assay, at day 7 after injury, the water content was significantly higher in the brains of E4 mice compared with E3 and WT mice. Additionally, a significantly higher water content was observed in the KO group than in the WT group. (*P < 0.05 vs WT mice and E3 mice; ^#^ *P* < 0.05 vs WT mice. N = 8). The data are presented as the mean ± SD.

The CCI operation led to more EB extravasation into the brain tissues in each group (Fig. 3A). No significant differences in BBB permeability were observed in any group of mice at day 1 or day 3 after injury. However, at day 7, increased EB dye extravasation was found in the brains of KO mice compared with WT mice (*P* < 0.01). Furthermore, as shown in Fig. 3A, increased EB dye extravasation was detected in the brains of E4 mice compared with E3 and WT mice at day 7 after injury (*P* < 0.05 vs E3 mice; *P* < 0.01 vs WT mice). However, no significant difference was observed between the E3 and WT groups (*P* > 0.05).

### Brain Water Content

Significant increases of water content in the injured hemispheres were detected by the dry/wet weight method at day 1, day 3 and day 7 after injury. As shown in Fig. 3B, at day 1 and day 3 after injury, no significant differences of water content were found among the four groups. However, at day 7 after injury, the brain water content was significantly higher in the KO mice than in the WT mice (*P* < 0.05). Additionally, compared with E4 mice, the brain water content was significantly lower in the WT and E3 mice (*P* < 0.05). No significant difference was found between the E3 group and the WT group (*P* > 0.05), indicating that brain edema was more severe in E4 mice than in WT and E3 mice after injury.

### Occludin and ZO-1 Expression at Day 7 after TBI

**Figure 4:**
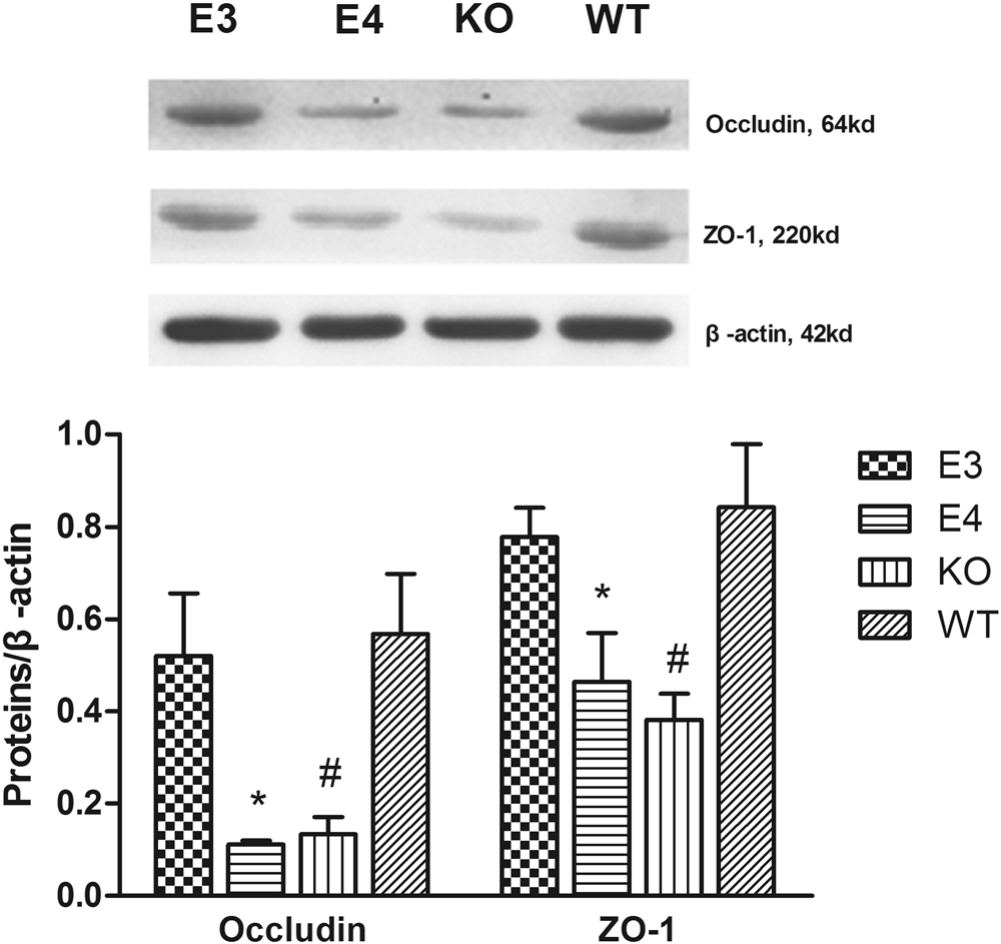
The Western blot results showed that Occludin and ZO-1 contents in the WT mice were significantly higher than those in the E4 and KO mice, and higher expressions were observed in E3 mice than in E4 mice. (*P < 0.05 vs E3 mice and *P* < 0.01 vs WT mice; ^#^ *P* < 0.01 vs WT mice. N = 8). The data are presented as the mean ± SD.

Various severities of neurological deficits and BBB damage were found among different transgenic mice at day 7, but not at day 1 or day 3, after injury. Therefore, at day 7 after injury, we detected the expressions of the main components of the BBB, such as the tight junction proteins, Occludin and ZO-1 (Fig. 4).

Tight junction proteins, Occludin and ZO-1, were detected at day 7 after injury by Western blot. The results showed that both Occludin and ZO-1 were obviously decreased in KO mice compared with WT mice (*P* < 0.01). Additionally, compared with E4 mice, E3 mice showed significantly higher levels of tight junction proteins in the injured cortex (*P* < 0.01 for Occludin; *P* < 0.05 for ZO-1). The results showed that the tight junction proteins, Occludin and ZO-1, were significantly more highly expressed in the injured cortex of WT mice than in KO or E4 mice. Compared with the E4 mice, higher expression was observed in the E3 mice.

### The Expression of NF-κB/MMP-9 at Day 7 after TBI

**Figure 5:**
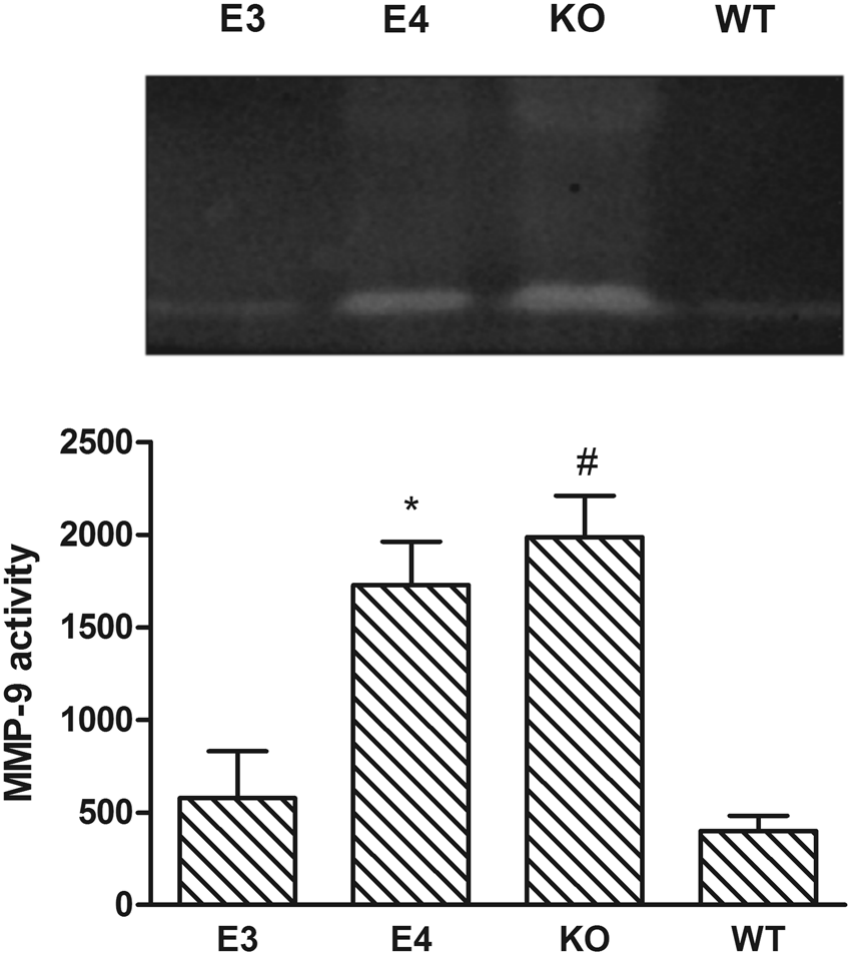
MMP-9 activity was detected at day 7 after injury and was significantly higher in the E4 mice compared with the E3 mice and the WT mice. More activity was found in the KO mice than in the WT mice. (*P < 0.01 vs E3 and *P* < 0.001 vs WT mice; ^#^ *P* < 0.001 vs WT mice. N = 8). The data are presented as the mean ± SD.

To explore the effects of different ApoEs on the NF-κB/MMP-9 pathway, the expression and activity of MMP-9 and NF-κB were studied in different transgenic mice. As shown in Fig. 5, MMP-9 activity was quantified using the gelatin zymography method at day 7 after injury in each group. Compared with WT mice, significantly more activated MMP-9 was detected in KO mice (*P* < 0.001). Furthermore, significantly less activated MMP-9 was found in E3 mice (*P* < 0.01 vs E4) and WT mice (*P* < 0.001 vs E4) compared with E4 mice, which indicated that more MMP-9 was activated in the E4 group than in the E3 and WT groups. However, no significant differences in MMP-9 activation were observed between the E3 and WT groups.

**Figure 6:**
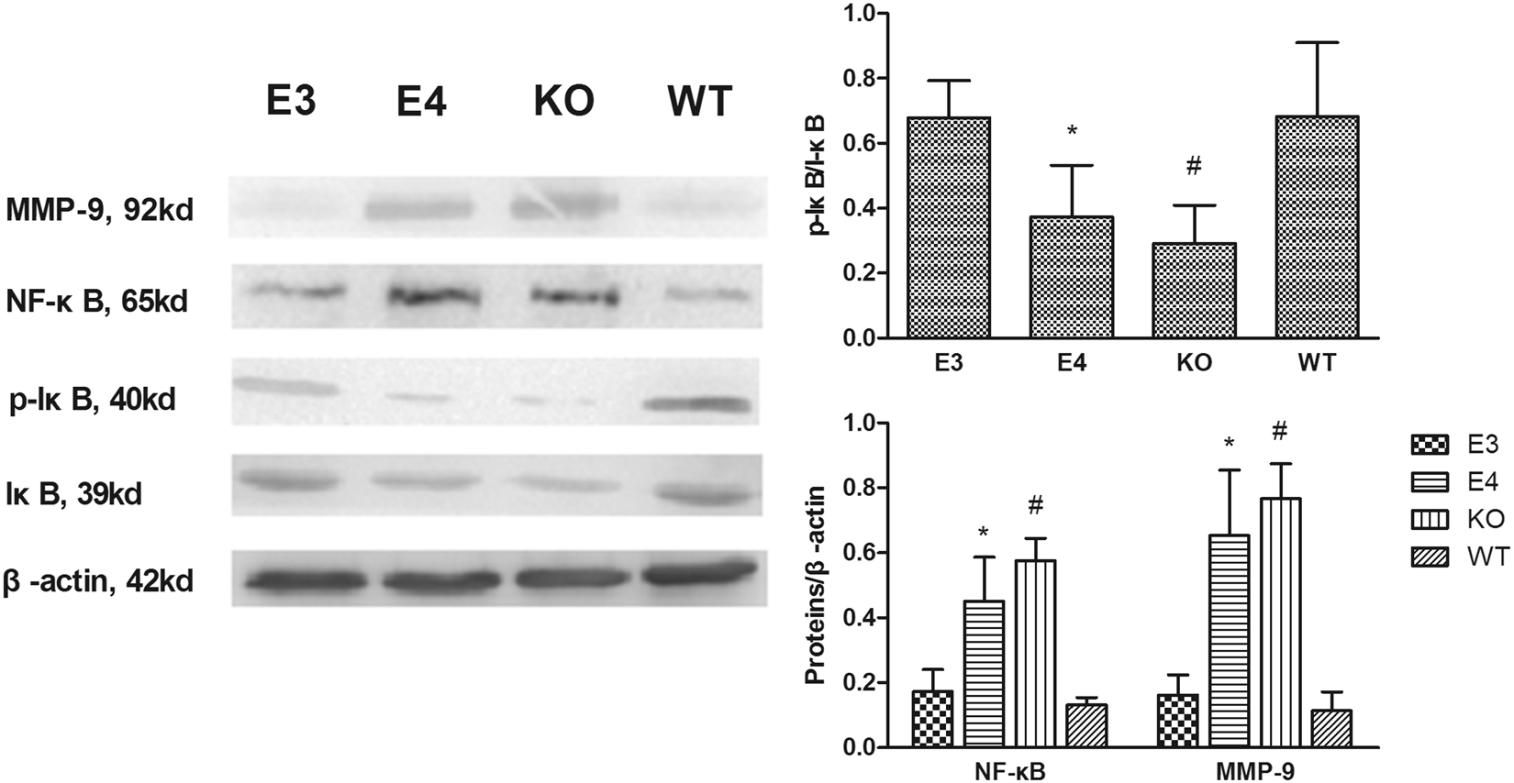
At day 7 after injury, I-κB phosphorylation and NF-κB suppression were significantly more obvious in E3 and WT mice compared with E4 mice. The KO mice presented less phosphorylation than the WT mice. (*P < 0.01 vs E3 and WT mice; ^#^ *P* < 0.01 vs WT mice. N = 8) The expression of NF-κB and MMP-9 was significantly higher in the E4 mice than in the E3 and WT mice. Additionally, more expression was found in the KO mice than in WT mice. (*P < 0.05 vs E3 and WT mice; ^#^ *P* < 0.001 vs WT mice. N = 8) The data are presented as the mean ± SD.

The expression of the NF-κB/MMP-9 pathway was detected by Western blot assay (Fig. 6). MMP-9 expression was significantly higher in the KO mice than in the WT mice at day 7 after injury (*P* < 0.01). Similarly, MMP-9 expression in the E4 mice markedly increased compared with E3 and WT mice (*P* < 0.05 both). Additionally, NF-κB expression obviously increased in the KO mice, with significantly decreased I-κB activation compared with WT mice (*P* < 0.01). Furthermore, significantly more activated I-κB and less NF-κB expression were detected in E3 and WT mice compared with E4 mice (*P* < 0.01 both).

## Discussion

The disruption of BBB integrity greatly influences the events after TBI, and this disruption is directly related to brain edema and neuro-functional impairment. Both ApoE and MMP-9 are considered to have impacts on BBB integrity. According to our recent study^23^, ApoE may influence MMP-9 activity and BBB integrity. The results of the current study showed that different ApoE isoforms have distinct impacts on MMP-9 and the BBB after TBI.

To study the possible mechanism through which ApoE influences the prognosis after TBI, the CCI model, a commonly used tool to simulate TBI, was conducted on E3 and E4 transgenic mice, ApoE knock-out mice, and WT mice in the present study. As shown by the Rotarod test and NSS results, severe neurological dysfunction was observed in the post-TBI mice of each group. At day 7, the neurological functions of E3 and WT mice were much better than that of the E4 and KO mice, indicating that neuro-function after TBI may be deteriorated by a lack of ApoE and the existence of ApoE4, which can be improved by ApoE3.

Similarly, severe BBB damage and brain edema were found in the mice of all groups at day 1 and day 3 after TBI, but no significant difference was found among the E3, E4, KO and WT mice. However, at day 7, compared with E3 and WT mice, the extravasation of Evans Blue dye increased and brain edema was more severe in the brains of KO and E4 mice, indicating that ApoE4 and a lack of ApoE lead to more severe disruptions of permeability and integrity than ApoE3 and WT-apoE. Therefore, at day 7 after injury, BBB damage and brain edema gradually eased in E3 and WT mice but not in E4 and KO mice, which was consistent with the results of neurobehavioral status after injury. BBB rupture is a basic pathophysiological change after TBI and an important mechanism of secondary brain injury^26^. Previous studies showed that a deficiency of ApoE may exacerbate brain edema after brain trauma by promoting BBB breakdown and increasing the injury susceptibility of the BBB^17, 22^, which demonstrates that ApoE may influence BBB integrity after TBI. In the present study, neurobehavioral status, BBB permeability and the brain water content of the four groups differed at day 7 after injury, and neuro-dysfunction, BBB damage and brain edema were significantly more severe in E4 mice than in E3 and WT mice. These data indicated that different ApoE isoforms had distinct effects on BBB integrity and neurological function after TBI. ApoE4 may deteriorate the neurological function of mice after TBI by aggravating the disruption of the BBB and brain edema, but ApoE3 may reduce BBB damage to improve the prognosis of TBI.

Tight junction proteins are important components of the BBB. After TBI, the expressions of tight junction proteins have been shown to be reduced, which was also confirmed in this study. To study the possible mechanism through which ApoE isoforms influence BBB integrity after TBI, the expressions of tight junction proteins, Occludin and zonula occluden-1 (ZO-1), were detected at day 7 after TBI in each group. As shown in Fig. 4, compared with E3 and WT mice, less tight junction proteins were detected in the brains of KO and E4 mice, which means that ApoE3, not ApoE4, protects the integrity of the BBB. The result suggested that ApoE influences tight junction proteins after TBI. ApoE4 might aggravate the disruption of BBB integrity by suppressing the expression of tight junction proteins, and ApoE3 might alleviate the disruption by improving their expression.

Previous studies have shown that ApoE may influence the BBB in AD^13, 27^, but different ApoE isoforms have different impacts. For instance, ApoE4 is considered to have increased the possibility of BBB breakdown, which is consistent with the results of the present study. However, how ApoE affects the BBB after TBI remains elusive. As the most important apolipoprotein in the brain, ApoE binds to all members of the low-density lipoprotein receptor (LDLR) family. As shown in previous studies, ApoE may influence tight-junction proteins and BBB integrity through low-density lipoprotein receptor-related protein-1 (LRP1), a member of the LDLR family^12, 21^. Furthermore, by binding to LRP1, ApoE may affect NF-κB-dependent MMP9 activation through cyclophilin A^13, 27^. MMP-9 is closely associated with BBB disruption and brain edema. According to previous studies, MMP-9 can be activated by NF-κB through the NF-κB/MMP-9 pathway in cerebral vessels, which leads to BBB breakdown^13, 28^.

To explore the impacts of different ApoE isoforms on NF-κB/MMP-9 after TBI, the activation of I-κB/NF-κB and MMP-9 was studied in different ApoE-genotype mice. The results of the present study showed that at day 7 after TBI, NF-κB expression was significantly higher in the brains of E4 and KO mice than in the brains of E3 mice and WT mice. Consistent with the expression of NF-κB, significantly more NF-κB was activated in the E4 and KO mice. Both the expression and the activity of MMP-9 in the brains of E4 and KO mice were significantly higher than in E3 mice and WT mice. These results indicated that after TBI, different ApoE isoforms have distinct impacts on the NF-κB/MMP-9 pathway. ApoE3 may inhibit the NF-κB/MMP-9 pathway by decreasing the expression and activation of NF-κB and MMP-9, but ApoE4 may promote the NF-κB/MMP-9 pathway by increasing such expression and activation. These data suggested that ApoE4, not ApoE3, may lead to BBB disruption by activating the NF-κB/MMP-9 pathway after TBI.

Three ApoE isoforms, ApoE2, ApoE3 and ApoE4, exist in the human brain. ApoE4 is considered a dysfunctional or harmful protein, which was confirmed in this study. Furthermore, this study showed that ApoE4 might increase the activation of the NF-κB/MMP-9 pathway to aggravate BBB disruption after TBI, which will eventually worsen the prognosis of TBI. Therefore, accurate methods that target ApoE4 gene carriers and the inhabitation or reduction of ApoE4-induced NF-κB/MMP-9 pathway activation may decrease the harmful effects of ApoE4 and improve the prognosis after TBI. Approaches that can both increase the neuro-protection of ApoE3 and decrease the neuro-impairment of ApoE4 may be promising therapies for all TBI patients. These speculations are being tested and verified in our further study.

In conclusion, the results of our study showed that different ApoE isoforms had distinct effects on neuro-function and BBB integrity after TBI. ApoE4 might worsen the outcomes of TBI by exacerbating the disruption of BBB integrity and activating the NF-κB/MMP-9 pathway. Moreover, the results of this study also provide evidence for the development of precise medications for TBI according to genotypes. Further studies are required to explore the therapeutic aspect of the mechanism and to identify a means to increase the protection by ApoE3 and decrease the impairment by ApoE4.

## Electronic supplementary material

Supplementary Information

## References

[1] Unterberg AW, Stover J, Kress B, Kiening KL (2004). Edema and brain trauma. Neuroscience.

[2] Rosenberg GA (1995). Matrix metalloproteinases in brain injury. J Neurotrauma.

[3] Jha R (2014). Fluid-attenuated inversion recovery hyperintensity correlates with matrix metalloproteinase-9 level and hemorrhagic transformation in acute ischemic stroke. Stroke; a journal of cerebral circulation.

[4] Batra A (2010). Increased plasma and tissue MMP levels are associated with BCSFB and BBB disruption evident on post-contrast FLAIR after experimental stroke. Journal of cerebral blood flow and metabolism: official journal of the International Society of Cerebral Blood Flow and Metabolism.

[5] Guo Z (2010). Matrix metalloproteinase-9 potentiates early brain injury after subarachnoid hemorrhage. Neurological research.

[6] Guo Z, Sun X, He Z, Jiang Y, Zhang X (2010). Role of matrix metalloproteinase-9 in apoptosis of hippocampal neurons in rats during early brain injury after subarachnoid hemorrhage. Neurological sciences: official journal of the Italian Neurological Society and of the Italian Society of Clinical Neurophysiology.

[7] Guo, Z., Xu, L., Wang, X. & Sun, X. MMP-9 expression and activity is concurrent with endothelial cell apoptosis in the basilar artery after subarachnoid hemorrhaging in rats. *Neurological sciences: official journal of the Italian Neurological Society and of the Italian Society of Clinical Neurophysiology*, doi:10.1007/s10072-015-2092-6 (2015). 10.1007/s10072-015-2092-6 25627354

[8] Chen YJ, Chang LS (2014). Simvastatin induces NFkappaB/p65 down-regulation and JNK1/c-Jun/ATF-2 activation, leading to matrix metalloproteinase-9 (MMP-9) but not MMP-2 down-regulation in human leukemia cells. Biochemical pharmacology.

[9] Lee CS (2009). New mechanism of rosiglitazone to reduce neointimal hyperplasia: activation of glycogen synthase kinase-3beta followed by inhibition of MMP-9. Arteriosclerosis, thrombosis, and vascular biology.

[10] Jia F (2014). MMP-9 inhibitor SB-3CT attenuates behavioral impairments and hippocampal loss after traumatic brain injury in rat. J Neurotrauma.

[11] Verghese PB, Castellano JM, Holtzman DM (2011). Apolipoprotein E in Alzheimer’s disease and other neurological disorders. The Lancet. Neurology.

[12] Halliday MR (2016). Accelerated pericyte degeneration and blood-brain barrier breakdown in apolipoprotein E4 carriers with Alzheimer’s disease. Journal of cerebral blood flow and metabolism: official journal of the International Society of Cerebral Blood Flow and Metabolism.

[13] Bell RD (2012). Apolipoprotein E controls cerebrovascular integrity via cyclophilin A. Nature.

[14] Jiang Y (2006). Effect of APOE polymorphisms on early responses to traumatic brain injury. Neuroscience letters.

[15] Cheng C (2015). Effect of APOE Gene Polymorphism on Early Cerebral Perfusion After Aneurysmal Subarachnoid Hemorrhage. Translational stroke research.

[16] Chen L, Sun X, Jiang Y, Kuai L (2015). APOEepsilon4 increases trauma induced early apoptosis via reducing delayed rectifier K(+) currents in neuronal/glial co-cultures model. Experimental cell research.

[17] Jiang L (2015). Effects of ApoE on intracellular calcium levels and apoptosis of neurons after mechanical injury. Neuroscience.

[18] Zhong J (2016). Bexarotene protects against traumatic brain injury in mice partially through apolipoprotein E. Neuroscience.

[19] Fullerton SM, Shirman GA, Strittmatter WJ, Matthew WD (2001). Impairment of the blood-nerve and blood-brain barriers in apolipoprotein e knockout mice. Experimental neurology.

[20] Hafezi-Moghadam A, Thomas KL, Wagner DD (2007). ApoE deficiency leads to a progressive age-dependent blood-brain barrier leakage. American journal of physiology. Cell physiology.

[21] Nishitsuji K, Hosono T, Nakamura T, Bu G, Michikawa M (2011). Apolipoprotein E regulates the integrity of tight junctions in an isoform-dependent manner in an *in vitro* blood-brain barrier model. The Journal of biological chemistry.

[22] Zhou S (2013). Apolipoprotein E protects astrocytes from hypoxia and glutamate-induced apoptosis. FEBS letters.

[23] Cao F (2016). Apolipoprotein E-Mimetic COG1410 Reduces Acute Vasogenic Edema following Traumatic Brain Injury. Journal of neurotrauma.

[24] Zheng M (2014). ApoE-deficient promotes blood-brain barrier disruption in experimental autoimmune encephalomyelitis via alteration of MMP-9. Journal of molecular neuroscience: MN.

[25] Flierl MA (2009). Mouse closed head injury model induced by a weight-drop device. Nature protocols.

[26] Ghajar J (2000). Traumatic brain injury. Lancet.

[27] Zlokovic BV (2013). Cerebrovascular effects of apolipoprotein E: implications for Alzheimer disease. JAMA neurology.

[28] Candelario-Jalil E (2011). Matrix metalloproteinases are associated with increased blood-brain barrier opening in vascular cognitive impairment. Stroke; a journal of cerebral circulation.

