# Supplementary material for: ApoE Influences the Blood-Brain Barrier Through the NF-κB/MMP-9 Pathway After Traumatic Brain Injury
Source: Sci Rep. 2017 Jul 27;7:6649. doi: 10.1038/s41598-017-06932-3 (PMC5532277; doi:10.1038/s41598-017-06932-3)
Supplement: Supplementary file 1 — Supplementary Information [file 41598_2017_6932_MOESM1_ESM.doc]

**Title Page**

**ApoE Influences the Blood-Brain Barrier Through the NF-κB/MMP-9 Pathway After Traumatic Brain Injury**

Zhipeng Teng^b^, Zongduo Guo^a^, Jianjun Zhong^a^, Chongjie Cheng^a^, Zhijian Huang^a^, Yue Wu^a^, Shuang Tang ^d^, Chao Luo^b^, Xing Peng^b^, Haitao Wu^c^, Xiaochuan Sun ^a^, Li Jiang^a^ *

Zhipeng Teng and Zongduo Guo contributed equally to this work.

^a^ Department of Neurosurgery, the First Affiliated Hospital of Chongqing Medical University

^b^ Department of Neurosurgery, Chongqing Traditional Chinese Medicine Hospital

^c^ Department of Neurosurgery, The Affiliated Hospital of Zunyi Medical College

^d^ Department of Neurosurgery, Suining Central Hospital

* Corresponding author: Li Jiang, MD

Department of Neurosurgery

The First Affiliated Hospital of Chongqing Medical University

No. 1, Youyi Road, Yuanjiagang,

Yuzhong District, Chongqing 400016,

PR China

Tel.: +86 23 89011152

Fax: +86 23 68734337

Email: 156313147@qq.com

**Supplementary Materials**

**Figure 1. The Western blot showed expression of ApoE in different mice.**

**
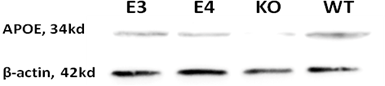
**

Table 1. Neurological Severity Scores (NSS) for Mice.

| **Task** | **Description** | **Points (success/failure)** |
| --- | --- | --- |
| Exit circle | Ability and initiative to exit a circle of 30 cm diameter within 3 min | 0/1 |
| Monoparesis/hemiparesis | Paresis of upper and/or lower limb of the contralateral side | 0/1 |
| Straight walk | Alertness, initiative and motor ability to walk straight | 0/1 |
| Startle reflex | Innate reflex; the mouse will bounce in response to a loud hand clap | 0/1 |
| Seeking behavior | Physiological behavior as a sign of ‘interest’ in the environment | 0/1 |
| Beam balancing | Ability to balance on a beam of 7-mm width for at least 10 s | 0/1 |
| Round stick balancing | Ability to balance on a round stick of 5-mm diameter for at least 10 s | 0/1 |
| Beam walk: 3 cm | Ability to cross a 30-cm long beam of 3-cm width | 0/1 |
| Beam walk: 2 cm | Same task, increased difficulty on a 2-cm wide beam | 0/1 |
| Beam walk: 1 cm | Same task, increased difficulty on a 1-cm wide beam | 0/1 |
| Maximal score |  | 10 |
